# Efficacy and safety of oral nifedipine with or without vaginal progesterone in the management of threatened preterm labor

**DOI:** 10.18502/ijrm.v17i9.5098

**Published:** 2019-09-22

**Authors:** Bushra Ashraf

**Affiliations:** Department of Obstetrics and Gynecology, Pakistan Institute of Medical Sciences (PIMS) Islamabad Pakistan.

**Keywords:** Preterm labor, Tocolytics, Nifedipine, Progesterone

## Abstract

**Background:**

Preterm labor (PTL) is a serious emergency wherein robust management is imperative for achieving improved outcome.

**Objective:**

To evaluate the efficacy and safety of nifedipine alone vs nifedipine with vaginal progesterone in managing threatened PTL.

**Materials and Methods:**

This comparative study was carried out at the Pakistan Institute of Medical Sciences, Islamabad over a 2-year’ period, from September, 2013 to August, 2015. The study included 276 patients with threatened PTL. Half of them were allocated to nifedipine alone group whereas the remainder half to the additional progesterone group. In nifedipine alone group (group A), all the patients were given 20░mg of rapid release nifedipine orally. If uterine contraction continued, a 10░mg dose was repeated every 20░min with a maximum of 40░mg within the first hour. After completing the first hour, 20░mg was given every 4–6 hr for 72░hr. In the additional vaginal progesterone group (group B), following successful tocolysis with nifedipine, additional - maintenance tocolysis was ensured with vaginal progesterone 200░mg daily.

**Results:**

Successful acute tocolysis was achieved with nifedipine among 86.23% patients. Mean pregnancy prolongation was 11.13░±░5.08 days in group A while 29.73░±░3.10 days in group B. (p░≤░0.001)

**Conclusion:**

Acute tocolytic therapy with nifedipine was successful in the majority of our patients. The additional daily use of vaginal progesterone suppositories resulted in significant prolongation of pregnancy as well as reduction in the rate of low birth weight and neonatal ICU admissions.

## 1 Introduction

As per the definition of the World Health Organization, Preterm labor (PTL) refers to the onset of labor after the gestation of viability and before 37 completed weeks of pregnancy (1,2). Compared to the actual PTL, a threatened PTL is diagnosed when there are documented uterine contractions without any evidence of cervical change. Spontaneous PTL and the consequent delivery account for approximately 50% of the preterm deliveries (PTDs), which in turn constitute the predominant causes of perinatal mortality and morbidity. The incidence of PTL is variably reported from different parts of the globe and ranges from 5% to 18% of all births (3–5). PTL has been a formidable foe for the healthcare professionals, both in the developing as well as developed countries. It is widely recognized that its prevention or effective management has the potential to improve the neonatal outcome and reduce the cost of management. A variety of pharmacological agents have been tested in the past to relax the uterine myometrium and so inhibit uterine contractions and stop the threatened PTL. For instance, nifedipine, magnesium sulphate, β-receptor agonists, prostaglandin synthase inhibitors, and oxytocin receptor blockers,etc (6–9).

The present study was carried out to determine the efficacy and safety of oral nifedipine with or without vaginal progesterone in the management of threatened PTL in terms of pregnancy prolongation, neonatal outcome and any maternal side effects of the therapeutic agents employed.

## 2 Materials and Methods

This quasi-experimental study was carried out at the Department of Obstetrics and Gynecology, Pakistan Institute of Medical Sciences, Islamabad over a period of two years, from September, 2013 to August, 2015. The study included all those patients with threatened PTL (i.e., between 24 and 37 completed weeks of gestation) who were presented with one or more of the following: (1) Four uterine contractions per 20░min, (2) Cervical dilatation of 0–3░cm in nulliparous and 1–3░cm in multiparous with intact membranes, and (3) Changing cervical effacement to 50% as observed on serial examinations. Exclusion criteria were patients with cervical dilatation of> 4░cm and ruptured membranes, non-reassuring fetal status, intrauterine growth restriction, congenital anomalies, any known maternal systemic disorders, and any contraindication to nifedipine therapy.

All patients were admitted for management indoor. They were assessed initially by detailed history, thorough examination, and baseline investigations. Half of the patients were randomly assigned to nifedipine alone group and the remaining half were randomized to the additional progesterone group. Computer generated random number table was employed for randomization. The two groups were matched for important initial demographic and clinical variables. A birth weight below the 5^th^ percentile was considered as intrauterine growth restriction. Patients with gestational age < 34 wk were given intramuscular injections of 6░mg dexamethasone, repeated every 12░hr for four consecutive doses. Intravenous injections of amoxicillin 1░g░m twice daily for 72░hr were also given to them.

### 2.1 The treatment protocol for each group was as follows

In Nifedipine alone group (group A), all the patients were given 20░mg of rapid release nifedipine orally. If uterine contractions continued, repeat dose of nifedipine capsule of 10░mg was given every 20░min. The maximum dose of nifedipine was limited to 40░mg within the first hour of starting the tocolytic treatment. Once the first hour was over, a regular dose of 20░mg nifedipine was administered every 4–6 hr consecutively for 72░hr. Dosage schedule was modified according to the patient’s clinical symptoms and vital signs. The blood pressure was monitored serially every 15░min to prevent hypotension.

Tocolysis was considered successful if uterine activity reduced to <4 contractions per hour with the absence of cervical change. Tocolytic failure was defined as delivery in <48░hr after the initiation of the therapeutic agents. Those with tocolytic failure were excluded from further analysis. In the additional vaginal progesterone group (group B), following successful tocolysis with nifedipine, the patients were treated with additional maintenance tocolysis with vaginal progesterone 200░mg daily until delivery or the 37^th^ completed wk of gestation.

The primary outcome measure analyzed was the prolongation of pregnancy (in terms of delivery delayed by 48^hr^ following acute tocolysis with nifedipine, delivery before 34 and 37 wk with maintenance tocolysis with vaginal progesterone). The secondary outcome measures included the neonatal outcome and any maternal side effects with the therapeutic agents employed.

### 2.2 Ethical consideration

The study protocol was approved by the hospital ethics committee (HEC) and written informed consent was taken from all the participating patients. Anonymity of the participants was ensured.

### 2.3 Statistical analysis

The data were recorded on the proforma and subjected to statistical analysis, using SPSS software (Statistical package for social sciences, Version 17.0, Chicago, IL, USA). The percentages of various outcome variables were compared by employing chi-square test and p░≤░0.05 was regarded as statistically significant.

## 3 Results

### 3.1 Demographic characteristics of the included patients

There were a total of 276 patients, with 138 patients in either group. There was no significant difference in the baseline maternal demographics and clinical characteristics of the two groups. The age of the patients ranged between 16 and 45░yr with a mean age of 23.03░±░8.35░yr in group A and 23.16░±░8.37░yr in group B. Majority of the patients (n░=░208;75.36%)presenting with threatened PTL were below 25 yrof age.

### 3.2 Presenting clinical features among the patients

Most of the patients (n░=░263; 95.28%) were presented to the emergency department while the remaining (n░=░13;4.71%) were presented to the outpatient department. The gestational age at presentation varied from 24 to 36 wk with a mean of 32.01░±░1.92 wk in group A while 32.07░±░1.81 wkin group B. The minimum gestational age at study entry was 24 wk and the maximum age was 36 wk.The important presenting clinical features such as the maternal age (yr), gestational age at presentation (wk), duration of the present threatened PTL, gravidity status (primigravida versus multigravida), singleton versus twin pregnancy, past history of PTL, past history of abortion are all summarized in table I.

**Table I:** The presenting features among the included patients. (n░=░138 each group)

**Parameters**	**Group A**	**Group B**
Maternal age^*^	23.03░±░8.35	23.16░±░8.37
Initial gestational age^*^	32.01░±░1.92	32.07░±░1.81
Duration of the present PTL^*^	6.57░±░5.62	6.77░±░5.90
Gravidity status (primigravida versus multigravida	113/ 25	112/26
Singleton versus twin pregnancy	127/11	126/12
Past PTL history^**^	17(12.31)	16(11.59)
Past history of abortion^**^	7(5.07)	6(4.34)

* Data presented as mean░±░SD. ^**^ Data presented as n(%)

The two groups were matched with respect to the baseline maternal demographics and clinical characteristics

PTL: Preterm labor

### 3.3 Pregnancy outcome data

Successful acute tocolysis with nifedipine was achieved in 86.23% of the initially recruited patients. There was significant prolongation of pregnancy/ the mean time of delivery postponement in the group B as compared to the group A. Mean pregnancy prolongation was 11.13░±░5.08 days in group A and 29.73░±░3.10 days in group B. (p░=░0.002) (Table II).

**Table II:** The pregnancy outcome measures. (n░=░138 each group)

**Parameters**	**Group A**	**Group B**	**p-value**
Time of postponing delivery (days)	11.13░±░5.08	29.73░±░3.10	≤0.001^*^a
Preterm birth before 34 weeks^**^	14(10.14)	8(5.79)	0.50^**^b
Preterm birth before 37 weeks^**^	73(52.89)	49(35.50)	≤0.001^*^a

* Data presented as mean░±░SD ^**^ Data presented as n(%)

* a p-value< 0.05 Significant ^**^ b p-value >0.05 Insignificant

### 3.4 Neonatal outcomes data

Table III shows the comparison of various neonatal outcome measures between the two groups of patients. The number of neonates admitted in neonatal intensive care unit and rate of Low birth weight were significantly less in group B than group A. (p░=░0.002 and p░≤░0.001 respectively). There was no case of intrauterine death.

**Table III:** The neonatal outcome measures (n░=░138 each group)

**Parameters**	**Group A**	**Group B**	**p-value**
Number of neonatal deaths	13(9.42)	3(2.17)	0.30^**^
LBW (Kg)	73(52.89)	31(22.46)	0.00^*^
RDS	19(13.76)	9(6.52)	0.50^**^
Number of neonates admitted in NICU	31 (22.46)	7(5.07)	0.00^*^
Apgar score <7	9(6.52)	2(1.44)	0.53^**^
Intra cranial bleeding	3(2.17)	-	1.00^**^
Sepsis	7(5.07)	2(1.44)	0.67^**^

Data presented as n(%) ^*^ p-value< 0.05 Significant, ^**^ p-value>0.05 Insignificant.

NICU: Neonatal intensive care unit LBW: Low birth weight RDS: Respiratory distress syndrome.

### 3.5 Maternal side effects of nifedipine

Hypotension 52(18.70%) and tachycardia 20(7.19%) were the commonest side effects observed with nifedipine therapy. There was no case of serious side effects that warranted discontinuation of the therapy (Table IV).

**Table IV:** The maternal side effects observed with nifedipine treatment (n░=░138 each group)

**Parameters**	**Group A**	**Group B**
Tachycardia	11(7.97)	9(6.52)
Hypotension	25(18.11)	27(19.56)
Headache	9(6.52)	11(7.97)
Hot flushes	5(3.62)	2(1.44)
Nausea/ vomiting	2(1.44)	0

Data presented as n(%)

There was no case of serious side effects that warranted discontinuation of the therapy

## 4 Discussion

In the present study, threatened PTL was more commonly observed among women of younger age groups (i.e. below 25 yrof age; 75.36%). Majority of them wereprimigravida (n░=░225; 81.52%). Our findings are similar to those reported by Ragunath and co-worker (10) from India, Klauser and co-worker (11) and Sharami and co-worker (12) from Iran. In the present study the mean gestational age at presentation was 32.01░±░1.92 wk in group A and 32.07░±░1.81 in group B patients. The mean gestational ages reported by Moramezi and co-worker (7), Sharami and co-worker (12), Gargari and co-worker (13) and Kashanian and co-worker (14) were 32░±░3 wk, 33.95░±░1.49 wk, 32.2░±░2.8 wk and 30.8░±░2.5 wk respectively. In our study we employed nifedipine for acute tocolysis as this medication is considered as the first-line tocolytic agent for the management of threatened PTL, given its established superiority over β-2 receptors agonists and magnesium sulfate. The tocolytic agentsdonot directly improve the neonatal outcomes. They rather delay the preterm delivery and thus provide an opportunity to administer corticosteroids to the mother and also ensure her transportation to a tertiary care facility for better neonatal care after delivery. In premature neonates, antenatal corticosteroids reduce morbidity and mortality. Tocolytic therapy therefore holds the potential to improve outcomes from preterm delivery in these cases. The preterm babies (born between 20 and 36 completed weeks of gestation) are generally more prone to be ill and are less likely to survive than babies born at term. The earlier these babies are born the more likely they are to have problems particularly because of their lung immaturity. The use of nifedipine reduces the risk of delivery within 7 days of initiation of treatment and delivery before 34 wk of gestation. This in turn helps to improve clinically important neonatal outcomes. For instance, respiratory distress syndrome, intraventricular hemorrhage, necrotizingenterocolitis, and neonatal jaundice (1–4,14).

In the present study, successful acute tocolysis with nifedipine was achieved in 86.23% of the initially recruited patients. Nifedipine successfully suppressed threatened labor when dilatation was less than 1.5░cm and effacement less than 50%. The mean prolongation of pregnancy was 11.13░±░5.08 days with nifedipine alone in the present study. Our findings conform to several reported studies on the use of nifedipine for acute tocolysis (10,11,14). Hypotension and tachycardia were the most common side effects observed with nifedipine in our study. The published literature has documented a variety of occasional serious adverse effects with nifedipine therapy. For instance, myocardial infarction, severe maternal dyspnea, pulmonary edema, maternal hypoxia, severe maternal hypotension with fetal death, and atrial fibrillation. A case series study reported that six out of seven cases of nifedipine-associated severe maternal dyspnea occurred in women with twin pregnancies and recommended to exercise caution when using nifedipine among patients with compromised cardiovascular function. The overall incidence of serious adverse effects reported with the use of nifedipine is ≤1%. Nifedipine does not have effects on fetal and neonatal death(10,15–17).

In our study the use of additional vaginal progesterone after successful tocolysis with nifedipine resulted in significant prolongation of pregnancy. It also resulted in reducing neonatal ICU admissions as well as the rate of LBW among the neonates. The importance of progesterone in maintaining pregnancy has long been recognized. Progesterone is a multifaceted hormone which has several crucial functions during pregnancy. For instance, it supports uterine quiescence, suppresses contractile genes and prevents the rejection of the fetus by the mother through suppressing the cellular component of the immune system. Despite the apparent benefits of progesterone in high-risk populations, progesterone has largely been studied only as a prophylactic method in asymptomatic women, not as a tocolytic agent in women with symptoms of threatened PTL. One study has shown that patients who remained undelivered after an episode of PTL underwent progressive cervical shortening during the three-week observational period and treatment with a high-dose progesterone was associated with both a lower cervical shortening as well as a reduced rate of PTB. Our finding of the positive effects of vaginal progesterone on significantly prolonged pregnancy conforms to several published studies from different parts of the world (12,13,18–20).

### 4.1 Strengths and limitations of the study

Our study has certain strengths as well as suffers some limitations. The major strength of the present study is that it was a well-designed quasi-experimental study performed for the first time in an institution in Pakistan. Second strength of this study is its apparent external validity, supported by the fact that our primary results are consistent with those of similar trials. Third strength of the study is the diverse nature of the Pakistani patient population presenting with variable demographic features. One limitation of the current study is that the primary endpoint (i.e. prolongation of pregnancy) was a surrogate for several neonatal outcomes. Secondly our case volume was not large enough to carry out in-depth analyses of the risk factors for various outcomes measures under scrutiny. Thirdly our study was limited to a single institution. Additionally there was no blinding or use of any placebo in the study to decrease any potential bias. Future well-designed local multicenter studies are recommended to improve upon the limitations of the present study.

## 5 Conclusion

Acute tocolytic therapy with nifedipine was successful in the majority of our patients without any serious untoward effects that warranted discontinuation of the medication. The additional daily use of vaginal progesterone suppositories of 200░mg (following successful acute tocolysis) was associated with a significant prolongation of pregnancy as well as significant reduction in the rate of LBW and neonatal ICU admissions.

## Conflict of Interest

The author declares to have no competing interests or other interests that could bias the results and discussion reported in this paper.
